# Genome mining of *Lactiplantibacillus plantarum* PA21: insights into its antimicrobial potential

**DOI:** 10.1186/s12864-024-10451-7

**Published:** 2024-06-06

**Authors:** Sharleen Livina Isaac, Ahmad Zuhairi Abdul Malek, Nurul Syafika Hazif, Farah Syahrain Roslan, Amalia Mohd Hashim, Adelene Ai-Lian Song, Raha Abdul Rahim, Wan Ahmad Kamil Wan Nur Ismah

**Affiliations:** 1 Department of Microbiology, Faculty of Biotechnology and Biomolecular Sciences, Universiti Putra Malaysia (UPM), Serdang, 43400 Selangor Malaysia; 2 Halal Products Research Institute, Universiti Putra Malaysia (UPM), Serdang, 43400 Selangor Malaysia; 3 Department of Cell and Molecular Biology, Faculty of Biotechnology and Biomolecular Sciences, Universiti Putra Malaysia (UPM), Serdang, 43400 Selangor Malaysia; 4 National Institutes of Biotechnology Malaysia (NIBM), Serdang, 43400 Selangor Malaysia

**Keywords:** *Lactiplantibacillus plantarum*, Postbiotic, Antimicrobial, Antimicrobial resistance, Genome mining

## Abstract

**Background:**

The dramatic increase of antimicrobial resistance in the healthcare realm has become inexorably linked to the abuse of antibiotics over the years. Therefore, this study seeks to identify potential postbiotic metabolites derived from lactic acid bacteria such as *Lactiplantibacillus plantarum* that could exhibit antimicrobial properties against multi-drug resistant pathogens.

**Results:**

In the present work, the genome sequence of *Lactiplantibacillus plantarum* PA21 consisting of three contigs was assembled to a size of 3,218,706 bp. Phylogenomic analysis and average nucleotide identity (ANI) revealed *L. plantarum* PA21 is closely related to genomes isolated from diverse niches such as dairy products, food, and animals. Genome mining through the BAGEL4 and antiSMASH database revealed four bacteriocins in a single cluster and four regions of biosynthetic gene clusters responsible for the production of bioactive compounds. The potential probiotic genes indirectly responsible for postbiotic metabolites production were also identified. Additionally, in vitro studies showed that the *L. plantarum* PA21 cell-free supernatant exhibited antimicrobial activity against all nine methicillin-resistant *Staphylococcus aureus* (MRSA) and three out of 13 *Klebsiella pneumoniae* clinical isolates tested.

**Conclusion:**

Results in this study demonstrates that *L. plantarum* PA21 postbiotic metabolites is a prolific source of antimicrobials against multi-drug resistant pathogens with potential antimicrobial properties.

**Supplementary Information:**

The online version contains supplementary material available at 10.1186/s12864-024-10451-7.

## Background

Antimicrobial resistance (AMR) is becoming more prevalent and continues to intimidate the ability to treat bacterial infections effectively. The reckless use of antibiotics has led to this very day where the potency of customary antimicrobials is diminishing due to the emergence of multi-drug resistant (MDR) bacteria. Methicillin resistant *Staphylococcus aureus* (MRSA) and *Klebsiella pneumoniae* are among clinically important MDR bacteria that are not only confined to healthcare settings but are also increasingly observed in the community [1, 2]. The World Health Organization (WHO) [3] attests that if this predicament is left unaddressed, AMR could lead to the resurgence of a pre-antibiotic era, where even the most basic infections can become fatal. Therefore, the emphasis on the discovery of new antimicrobial pipelines to tackle AMR and prevent a global health crisis is mandatory.

Lactic acid bacteria (LAB), in particular *Lactiplantibacillus plantarum* (previously known as *Lactobacillus plantarum*) [4], has been a subject of tremendous research lately due to its potential reservoir of novel antimicrobial compounds. This is largely due to its distinct properties which are attributed to a combination of factors such as the production of organic acids and bioactive compounds such as exopolysaccharides and bacteriocins [5]. Moreover, recent studies have highlighted the potential of *L. plantarum* as a candidate for the development of novel postbiotics, referring to bioactive compounds that are generated through the metabolic fermentation activity of LAB [6, 7]. The concept of postbiotics is particularly tempting because it single-handedly challenges the orthodox approach of purifying single compounds as analeptics. Postbiotics presents the possibility of harnessing the synergistic effects of multiple bioactive compounds which may offer a better therapeutic potential than any individual compound. Unlike previous reports on prebiotics and probiotics which are typically associated with live bacteria, postbiotics also offer a rather intriguing and safer alternative to combat AMR especially in immunocompromised individuals [6, 8].

Bioprospecting for postbiotic metabolites through the identification of biosynthetic gene clusters (BGCs) is one strategy to tap into the vast diversity of bacterial bioactive compounds. BGCs are modular gene units that work together to produce specific metabolites [9]. Advancements in genomics have facilitated a deeper understanding of how bacteria can be further exploited as a potential solution to AMR. Genome mining is such a tool that utilises bioinformatics to analyse bacterial genomes for the presence of BGCs and pathways that are involved in the production of bioactive compounds [10]. The search for antimicrobial compounds from *L. plantarum* PA21, in parallel with the advancements in genomics envisages a promising and swift avenue for the exploration of novel compounds in fighting AMR associated infections.

In this study, the phylogenetic relationship, and species classification of *L. plantarum* PA21 against other *L. plantarum* genomes was inferred. In addition, the whole-genome sequence of *L. plantarum* PA21 was mined to its potential antimicrobial compound while the probiotic features contributing to the antimicrobial activity was identified using the genome information. Subsequently, the bacterial inhibitory action of the antimicrobial substance from *L. plantarum* PA21 against MRSA and *K. pneumoniae* were investigated. The foundation laid on its genomic basis is expected to contribute to the overarching goals of developing targeted natural antimicrobial agents.

## Results

### Genome assembly and gene prediction

The gene prediction and annotation of *L. plantarum* PA21 were made using PATRIC (Supplementary Figure S1). The genome characteristics of *L. plantarum* PA21 are summarised in Table 1. The genome assembled into three contigs with N50 of 1,157,435. The genome size of 3,218,706 bp upon annotation was predicted to contain 3,118 protein coding sequences (CDS), 69 tRNAs, 14 rRNAs and 63 repeat regions. No plasmids were detected in the sequenced genome.

**Table 1:** The genome characteristics of *L. plantarum* PA21

Genome characteristics	Value
Contigs	3
Genome content	44.62%
Plasmids	0
Genome length	3,218,706 bp
Contig N50	1,157,435
Protein coding sequence (CDS)	3,118
Transfer RNA (tRNA)	69
Ribosomal RNA (rRNA)	14
Repeat regions	63
Partial CDS	0
Miscellaneous RNA	0

### Distribution of *L. plantarum* in environmental habitats

**Fig. 1:**
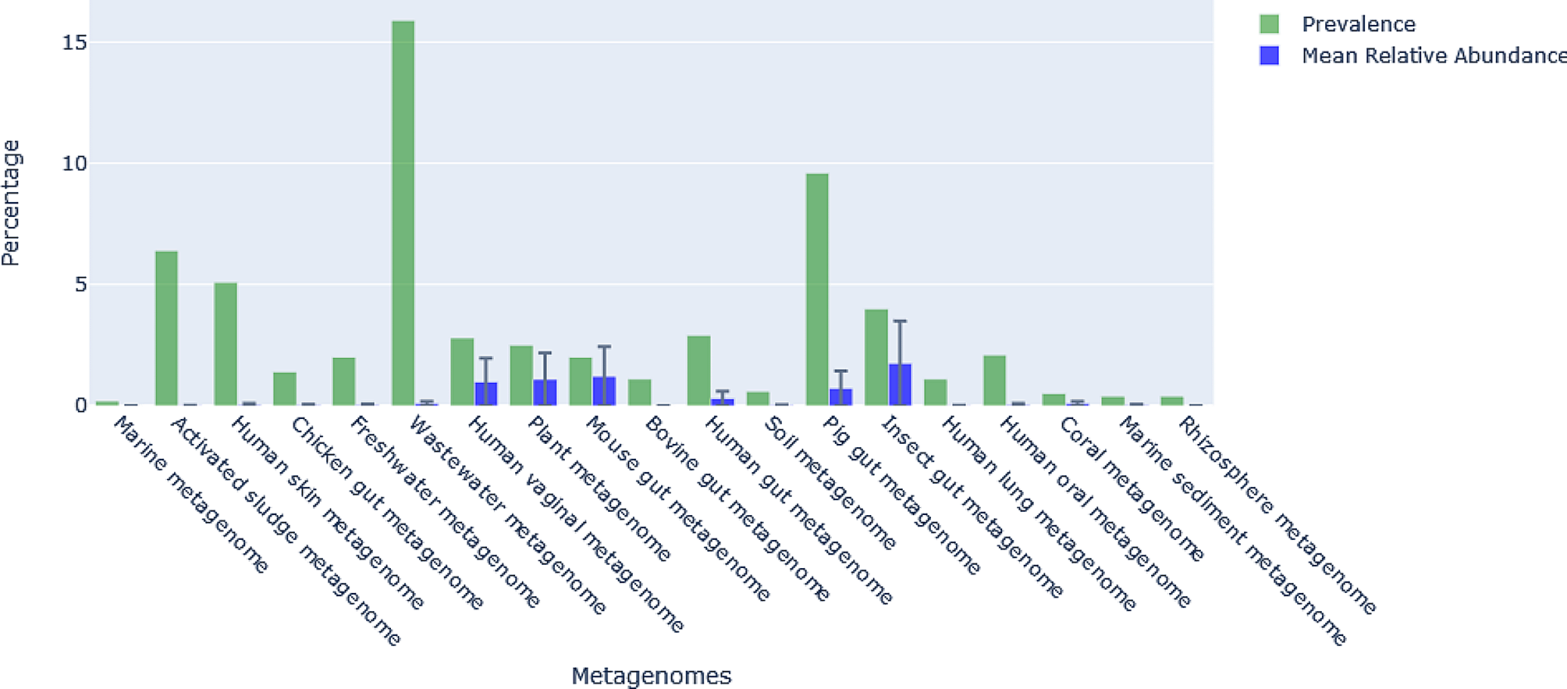
The prevalence and mean relative abundance of *L. plantarum* across different environments

*L. plantarum* was detected in a variety of environments ranging from gastrointestinal tracts of animals to freshwater and marine ecosystem (Fig. 1). Notably, high prevalence was detected in wastewater (15.90%) followed by pig gut (9.60%) and activated sludge (6.40%). The mean relative abundance throughout the environments was very low. The mean relative abundance of human, animal and insect gut metagenome displayed noticeable man relative abundance in general with high variability among samples with high standard deviation.

### Average nucleotide identity (ANI)

**Fig. 2:**
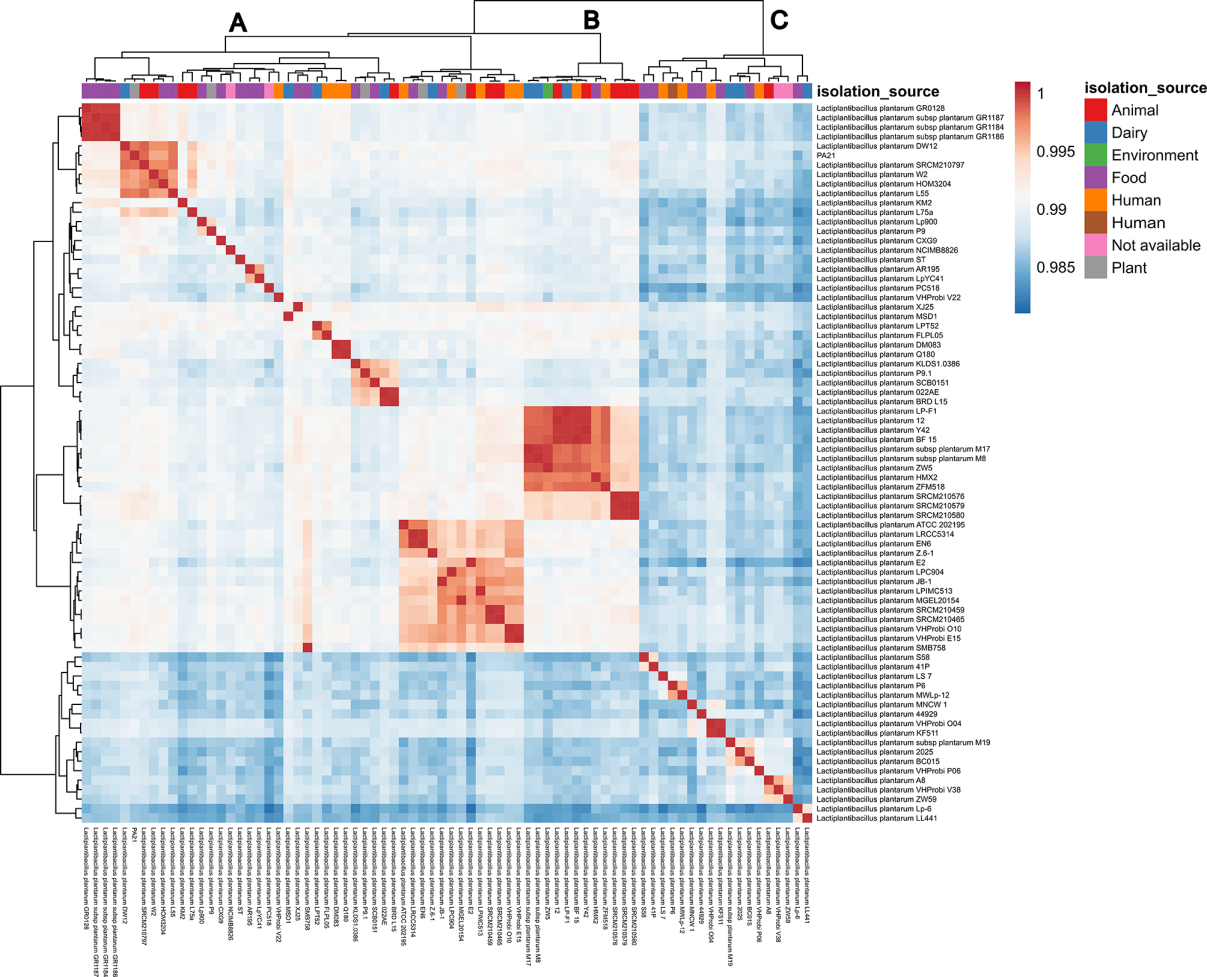
The heatmap depicts the average nucleotide identity (ANI) whole-genome comparison of other 75 *Lactiplantibacillus plantarum* genomes corresponding to its isolation sources

To investigate the genomic linkage and species boundaries, average nucleotide identity (ANI) analysis was undertaken on each *Lactiplantibacillus* genome assembly available from the NCBI database alongside *L. plantarum* PA21. The analysis of *L. plantarum* PA21 with other selected *Lactiplantibacillus* genomes revealed the formation of several clades through the heatmap clustering analysis of the ANI (Fig. 2). Based on the clustering, three distinct clades were identified. *L. plantarum* PA21 was found to be located in Clade A, specifically in a sub cluster consisting of other *L. plantarum* genomes originating from different isolation sources (Supplementary table S1). Notably, there are no patterns of clusters consists of similar isolation sources except for the first subcluster in Clade A which consist of genomes isolated from food-based sample namely, *L. plantarum* GR0128, *L. plantarum subsp plantarum* GR1184, *L. plantarum subsp plantarum* GR1186 and *L. plantarum subsp plantarum* GR1187.

### Phylogenomic analysis

**Fig. 3:**
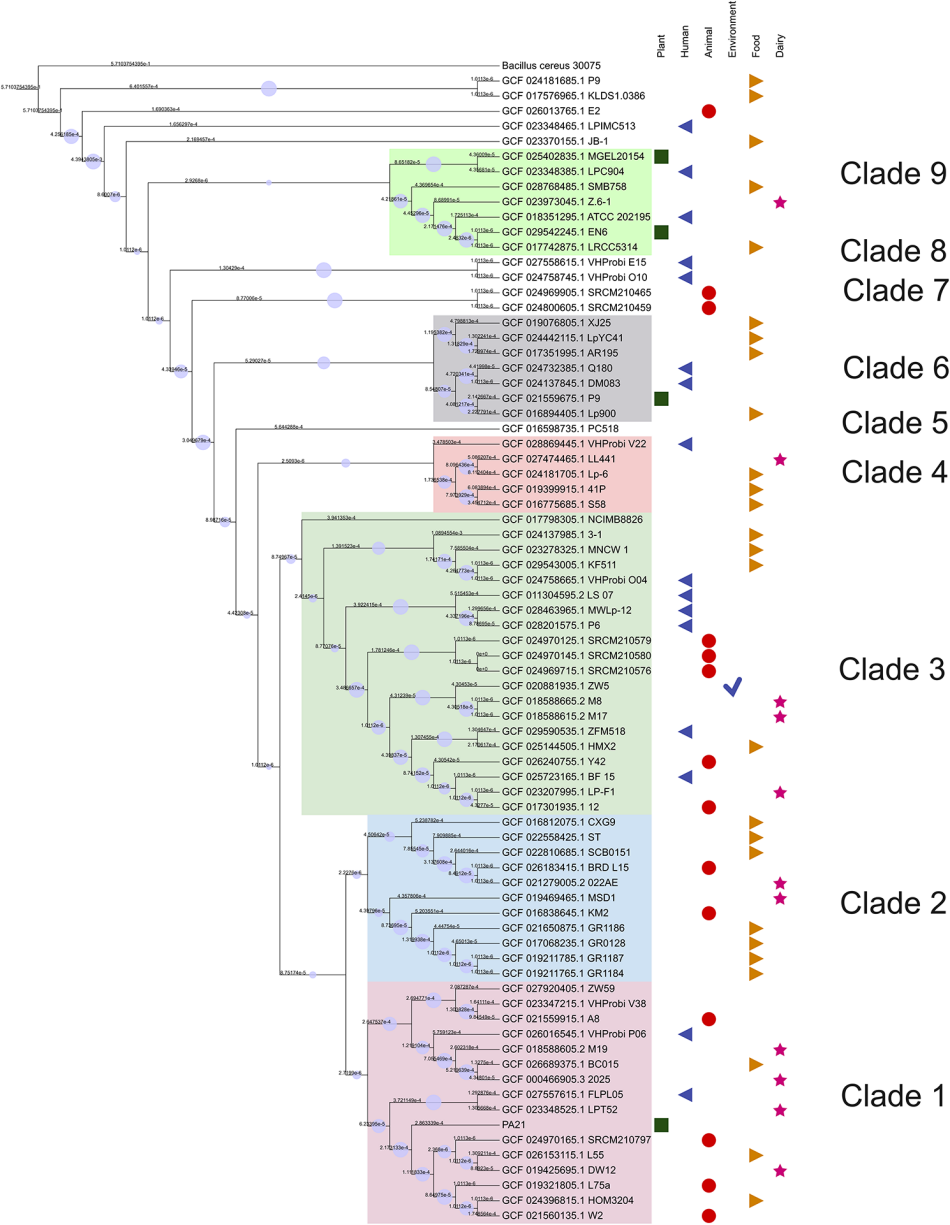
Phylogenomic maximum likelihood tree of 75 other *Lactiplantibacillus* genomes with *L. plantarum* PA21 and the outgroup, *Bacillus cereus* (accession: GCF_021655335.1). The branch lengths have been ignored and circled blue icon indicated bootstrap support for the depicted nodes in display. Binary data representing distinct shapes are used to denote the isolation source of the genome

To delve deeper into the evolutionary relationship of *L. plantarum* PA21 with other *Lactiplantibacillus* genomes, phylogenomic analysis was performed (Fig. 3). The phylogenetic tree grouped the genomes of *L. plantarum* into clusters based on taxonomic classification. The tree displayed the formation of three main large clades (clades 1–3). Interestingly, the taxonomic placement in the tree was not consistent with ANI’s genome placement.

### Pangenome analysis

**Fig. 4:**
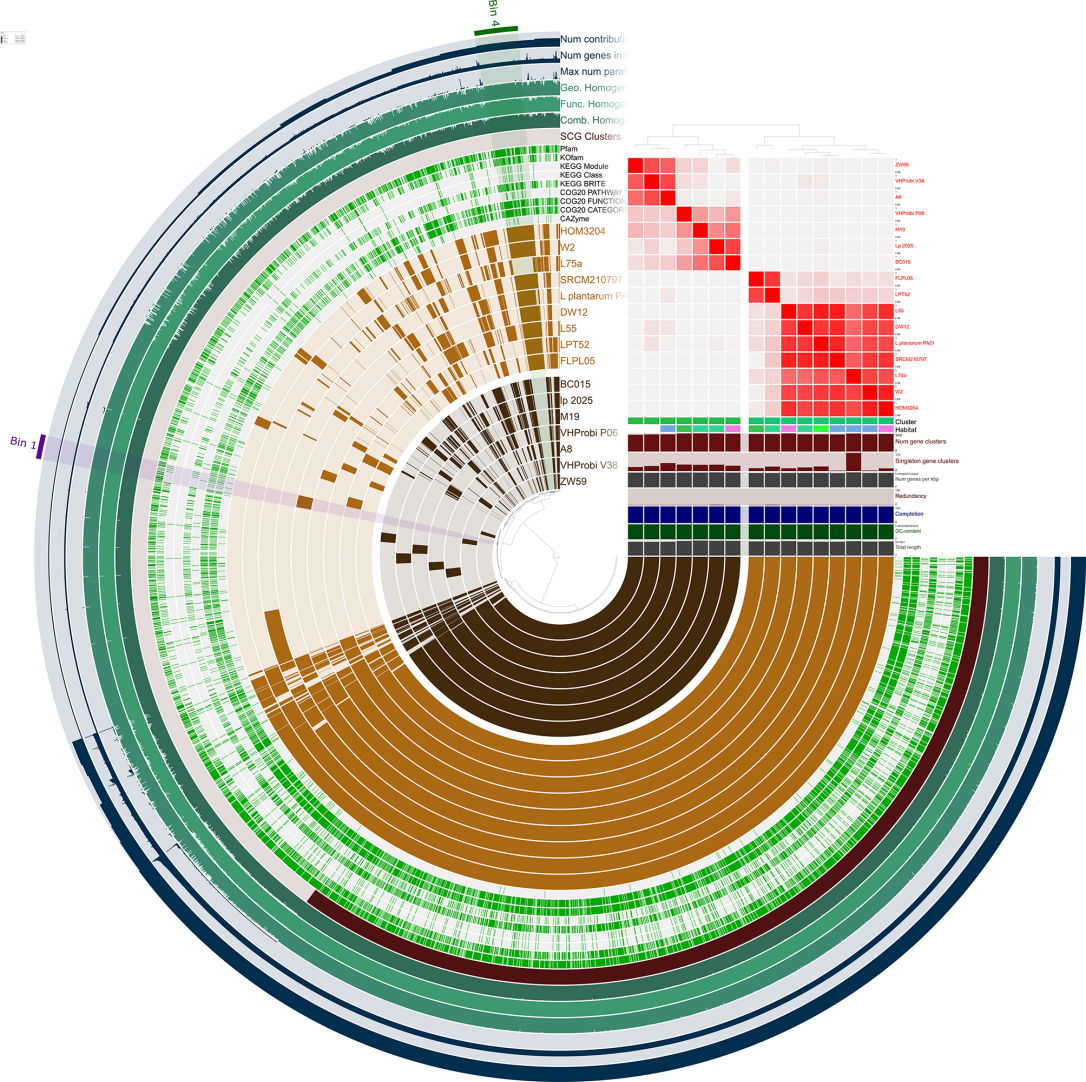
Pangenome plot of 16 genomes of *L. plantarum* generated through Anvio. Dark brown and light brown layer represent genomes from two clusters demarcated based on the ANI clustering (top right corner). Presence/absence of gene clusters represent by the presence/absence of dark/light brown box. The genomes are clustered based on the presence/absence of the gene clusters

Pangenome analysis of the genome (*n* = 16) clustered together in clade 1 resulted in 4559 gene cluster of which 785 gene clusters (17.21%) are singletons (only present in one genome), 2418 gene clusters (53%) are core gene cluster (exist in all 16 genomes) and 1356 gene clusters (29.74%) are accessory gene clusters (exist in 2–15 genomes) (Fig. 4). Out of 2418 core gene clusters, 2132 gene clusters are single copy core genes. Based on ANI clustering as shown, the 16 genomes can be separated into two cluster (cluster A – dark brown and cluster B – light brown). Through functional enrichment analysis between these clusters (Supplementary Table S4-S6), several genes were found to be significantly enriched in either cluster. Thorough inspection of the genes suggests that some of the genes involved in metabolism such as Keratan sulphate degradation, Nitrate assimilation, Dissimilatory nitrate reduction and Entner-Doudoroff pathway. As vividly shown in Fig. 2, the habitat distribution of the Clade 1 does not suggest the functional enrichment of these set of genes are due to habitat exclusivity. Analysis of the singletons found in *L. plantarum* PA21 revealed about 44 gene clusters solely present in *L. plantarum* PA21 where only 24 gene clusters are annotated while the other 20 gene clusters failed to be annotated by any of gene functional database employed in this study (Supplementary Table S3).

### Bacteriocin and secondary metabolite clusters gene prediction

**Fig. 5:**
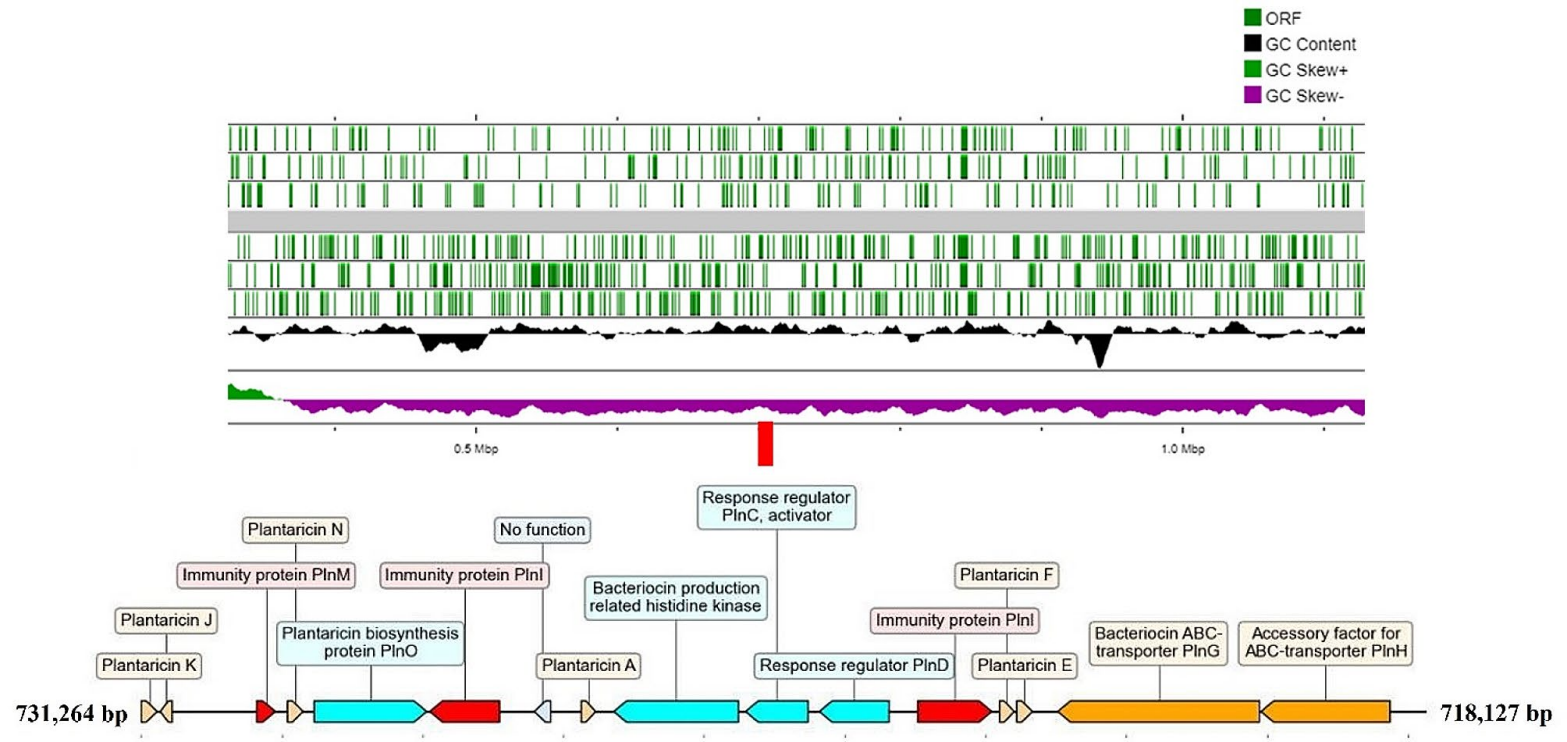
Bacteriocin gene cluster organisation and its localization on genome map

The genome information on *L. plantarum* PA21 was further investigated through bioinformatic analysis to gain genetic insights on their potential secondary metabolites. The antiSMASH and BAGEL4 server were used to screen for candidate BGCs. The screening revealed several BGCs that could encode antimicrobial compounds (Table 2). The *L. plantarum* PA21 was detected harbouring a single bacteriocin gene operon by BAGEL4 (Fig. 5) consisting of four bacteriocin structural genes (*plnJK, plnN, plnA* and *plnEF*). Further, two genes, HlyD and LanT were detected downstream of plantaricin E/F. Whereas, the antiSMASH database revealed four active metabolite regions.

**Table 2:** The genome-based identification of *L. plantarum* PA21 biosynthetic gene clusters (BGCs)

Location	Biosynthetic genes	Description	Software
Contig 1	Plantaricin_K	Bacteriocin class II with double-glycine leader peptide	BAGEL4
Contig 1	Plantaricin_J	-	BAGEL4
Contig 1	Plantaricin_N	Bacteriocin class II with double-glycine leader peptide	BAGEL4
Contig 1	Plantaricin_A	Alpha/beta enterocin family; Bacteriocin class II with double-glycine leader peptide	BAGEL4
Contig 1	Plantaricin_F	Lactococcin-like family; Bacteriocin class II with double-glycine leader peptide	BAGEL4
Contig 1	Plantaricin_E	-	BAGEL4
Contig 1	LanT	Bacteriocin ABC-transporter, ATP-binding and permease protein PlnG	BAGEL4
Contig 1	HlyD	Accessory factor for ABC-transporter PlnH	BAGEL 4
Region 3.1	Terpene	-	antiSMASH
Region 1.1	T3PKS	Type III polyketide synthase	antiSMASH
Region 2.1	Cyclic-lactone autoinducer	agrD-like cyclic lactone autoinducer peptides	antiSMASH
Region 2.2	RiPP-like	Other unspecified ribosomally synthesised and post-translationally modified peptide product	antiSMASH

### Probiotic genes and virulence-associated genes

The likelihood of probiotic-associated genes in *L. plantarum* PA21 responsible for adherence, immunomodulatory activity, stress, bile, and acid tolerance was analysed (Table 3). The genome screening revealed a palette of probiotic genes with similarity hits of more than 70%. These included *luxS, msa, lamA, srtA, clpC* and *dltB* genes. Also, as anticipated, no virulence genes were detected to be present in the genome by the VFDB database.

**Table 3:** Probiotic related genes in *L. plantarum* PA21

Gene	Function	GenBank ID	E-value	Similarity
Stress tolerance luxS	Production of autoinducer-2	ABC59818.1	5.00E-94	80.89%
*msrB*	Repair of oxidised protein	UFK68432.1	2.00E-70	69.93%
**Adherence** *fbp*	Fibronectin binding	AAV42987.1	2.00E-126	41.24%
*msa*	Adherence of bacteria to host cells	CCC78612.1	0.00E + 00	98.91%
*abpT*	Cell-surface anchor protein	ABE00714.1	0.00E + 00	69.42%
*lamA*	Cell-surface persistence protein	CCC80542.1	0.00E + 00	99.60%
*lspa*	Mucus-binding protein	ABD99635.1	2.00E-44	56.30%
*srtA*	Anchoring surface proteins to cell wall	CCC78006.1	0.00E + 00	100.00%
**Bile tolerance** *clpC*	Chaperone protein	CCC78435.1	0.00E + 00	100.00%
*lp_3160*	Multi-drug resistance transporter	CCC80194.1	2.00E-60	52.81%
**Immunomodulation**				
*dltD*	D-alanylation of teichoic acid	AXI93750.1	2.00E-121	49.76%%
*dltB*	D-alanylation of teichoic acid	QTO04092.1	0.00E + 00	100.00%
**Acid tolerance** *LBA1524-LBA1525*	Two-component regulatory system	AAV43343.1	3.00E-101	52.25%

### Antimicrobial activity of *L. plantarum* PA21

The antimicrobial activity of *L. plantarum* PA21 CFS was tested against nine MRSA and 13 *K. pneumoniae* clinical isolates. The inhibition zone and antimicrobial activity for all the tested strains were tabulated in Table 4. The highest antimicrobial activity was observed against *K. pneumoniae* 7839 (1373.8 mm^2^/mL). Yet, the CFS was only able to inhibit three out of 13 *K. pneumoniae* isolates. High antimicrobial activity was also observed against MRSA 10 (1202.4 mm^2^/mL). Overall, the effect of CFS was greater on MRSA isolates than on *K. pneumoniae* isolates. Following this, CFS-susceptible strains, MRSA 10, MRSA 14, MRSA 20, *K. pneumoniae* 6163, *K. pneumoniae* 7839 and *K. pneumoniae* 0926 were arbitrarily selected for subsequent tests.

**Table 4:** The antimicrobial activity of *L. plantarum* PA21 CFS against MDR isolates

Clinical isolates	*Diameter of inhibition zone (mm)	Antimicrobial activity (mm^2^/mL)	*Diameter of inhibition zone (mm) of positive control
Methicillin-resistant *Staphylococcus aureus*			
6	14.50 ± 0.00	1015.1	21 ± 0.00
7	14.00 ± 0.50	903.2	22 ± 0.00
8	12.50 ± 0.00	591.0	22 ± 0.50
10	15.33 ± 0.58	1202.4	25 ± 0.00
14	14.80 ± 0.58	1084.2	20 ± 0.50
16	12.00 ± 0.00	494.8	11 ± 0.00
17	11.00 ± 0.00	314.2	20 ± 0.00
19	11.00 ± 0.00	314.2	27 ± 0.00
20	14.67 ± 0.58	1053.5	24 ± 0.50
*Klebsiella pneumoniae*			
6163	14.50 ± 0.50	1015.1	19 ± 0.50
7839	16.00 ± 0.00	1373.8	20 ± 0.00
0926	11.00 ± 0.00	314.2	7 ± 0.50
1477	0.00 ± 0.00	0.0	17 ± 0.00
0770	0.00 ± 0.00	0.0	17 ± 0.00
1481	0.00 ± 0.00	0.0	8 ± 0.00
7293	0.00 ± 0.00	0.0	20 ± 0.00
1882	0.00 ± 0.00	0.0	25 ± 0.00
2942	0.00 ± 0.00	0.0	10 ± 0.00
8435	0.00 ± 0.00	0.0	24 ± 0.00
7951	0.00 ± 0.00	0.0	20 ± 0.00
7655	0.00 ± 0.00	0.0	16 ± 0.00
8207	0.00 ± 0.00	0.0	23 ± 0.00

*The inhibition zones are expressed in mm as the mean of 3 replicates, ± standard deviation

### Effect of pH, enzyme and temperature on *L. plantarum* PA21 CFS

The antimicrobial activity of *L. plantarum* PA21 CFS upon various pH, enzymes and heat treatments was analysed against MRSA 10 (Table 5). *L. plantarum* PA21 CFS completely lost its antimicrobial activity from pH 5 to pH 10, however, antimicrobial activity remained between pH 2 to pH 4 compared to untreated CFS. Interestingly, the antimicrobial activity of CFS was lost when treated with proteinase K and trypsin. However, the CFS of *L. plantarum* PA21 remained active at all tested temperatures with a slight decrease in its inhibitory effects at 121 °C.

**Table 5:** The effects of pH, enzyme and temperature on the antimicrobial activity of *L. plantarum* PA21 CFS

Treatment	*Diameter of inhibition zone (mm)
**Untreated CFS (Control)**	15.0
**pH**	
2	15.5
3	15.0
4	13.0
5	-
6	-
7	-
8	-
9	-
10	-
**Enzyme**	
Trypsin	-
Proteinase K	-
**Temperature (°C)**	
30	15.5
50	15.0
70	15.0
100	15.0
121	14.0

*MRSA 10 was used as the indicator strain and ‘-’ indicates no inhibition zone

## Discussion

The emergence of AMR in bacteria has by far been the biggest health challenge in modern medicine. With conventional antibiotics beginning to lose their potency against such pathogenic bacteria, natural antimicrobial compounds as alternatives are being greatly sought-after. Data from the current work suggests that *L. plantarum* PA21 postbiotic metabolites could be capitalised on as natural antimicrobial agents. The *L. plantarum* PA21 genome was first sequenced to understand its genetic makeup, identification, and function. In a comprehensive outlook, the genome size of *L. plantarum* PA21 (3.2 Mb) was within the range of most *L. plantarum* species (3.0 to 3.3 Mb) [11, 12]. Interestingly, the L. *plantarum* PA21 genome was devoid of a plasmid. Though it is common for most *L. plantarum* to carry one or more plasmids [13], some strains do not [14]. The absence of plasmid may have advantageous implications since it reduces the risk for horizontal gene transfer and the spread of antibiotic resistance genes to other bacteria. Also, this characteristic enables *L. plantarum* PA21 to be exploited for genetic manipulation studies.

Comparative genomic analysis employing phylogenomic tree inference and ANI provided valuable insights on the taxonomic status of the *L. plantarum* PA21 genome. The genome of interest, *L. plantarum* PA21 previously isolated from a tropical plant, was clustered together with other genomes of *L. plantarum* isolated from different isolation sources such as dairy, animal, and food. The analyses from this study were consistent with the findings from Liu et al. (2022) [15] whereby a wide genome association study of *L. plantarum* revealed that plant derived *L. plantarum* was dispersed relatively in the phylogenetic tree although the plant-based genome sample was small (*n* = 4) while ANI clustering corresponded with the three clades formation observation in a bigger sample size (*n* = 455). The clustering in this study, which is an uneven distribution of niche specific genomes in the phylogenetic tree, indicated a close evolutionary relationship between *L. plantarum* PA21 and other *L. plantarum* strains despite being isolated at different geographic locations and niches. Similar finding was also reported in the works of *Carpi* et al. (2022) [16] where each clade consisted of strain originates from multiple niches. Discrepancy between ANI and phylogenomic using single copy core genes can be attributed to the methodological differences. ANI measures overall genome similarity while the phylogenomic approach utilized the variation in the set of conserved genes. This study utilized pangenome analysis through Anvio of which each protein sequences from each selected genomes were blasted against one another and clustered into “gene cluster”. Large portion of the gene clusters were attributed to singletons (17%) suggesting the genomes of *L. plantarum* are quite adaptive with capacity to respond and evolve in response to diverse environmental conditions as depicted in the niche distribution of the *L. plantarum*. There are no patterns of gene cluster that are specific to niches as depicted in the works of *Leulier* et al. (2016) [17] suggesting *L. plantarum* have nomadic lifestyle.

There is ever-growing evidence that bacteriocins are among the bioactive compounds produced by *L. plantarum*, responsible for the inhospitable environment towards MDR bacteria [18, 19]. Thereby, the success of postbiotic metabolites in inhibiting MDR pathogens is potentially exemplified by the presence of bacteriocins. Genomic analysis of *L. plantarum* PA21 revealed the presence of four bacteriocins, akin to plantaricins (pln) in succession to genes encoding immunity proteins - common in most *L. plantarum*. The structural genes of *plnE* and *plnF* as well as *plnJ* and *plnK* placed adjacently to each other in the same operon operate based on a two-peptide bacteriocin system - *plnEF* and *plnJK*. In a classical study by Anderssen et al. (1998) [20], the complementary peptides identified from *L. plantarum* C11 were 1000 times more active against antagonistic strains when combined. The activation of *plnEF* and *plnJK* must first be preceded by *plnA* that interacts with the histidine protein kinase of a three-component regulatory system in the regulation of bacteriocin production. Nonetheless, with plnA acting as an ancillary peptide, little has been known about plnN which is purported to be a double glycine leader bacteriocin-like peptide [21]. Although the function of plnN was not explored further, its localization within the bacteriocin operon indicates the likelihood that it may be involved in bacteriocin regulation. Besides, the complete genome analysis also revealed the presence of BGCs such as terpenes, T3PKS, RiPP-like and cyclic-lactone inducers which are required for the synthesis of antimicrobial compounds. These BGCs were responsible for antagonising the growth of indicator strains, as documented in earlier investigations [22, 23]. So, the results of genome mining to some extent show that *L. plantarum* PA21 might have the potential to produce antimicrobial compounds.

Postbiotic metabolites are often the products of live bacterial metabolic processes, ergo the association of postbiotic metabolites to probiotic genes may likely be relevant [24]. Hence, a genome-based approach was adopted to evaluate the probiotic potential of *L. plantarum* PA21. Tolerance to stress, bile and acid is an important characteristic of any probiotic bacteria to weather through various unfavourable conditions. The physiological stress related genes in the *L. plantarum* PA21 genome included *luxS* and *clpC*. In *L. plantarum*, *luxS* gene has been an integral part in the biosynthesis of autoinducer-2 (AI-2) signalling molecules that mediates interspecies communication. Whilst commonly associated with stress tolerance, studies have also shown the substantial role of *luxS* in adhesion, acid and bile stress [25]. Most notably, is the ability to regulate bacteriocin production. The deletion of *luxS* altered the metabolic pathways thus affecting bacteriocin production in *L. plantarum* KLDS.0391 [26]. The *clpC* gene on the other hand is a member of regulatory ATPases implicated in stress tolerance and response. The response is effectuated through the ability to restore protein function as well as target misfolded proteins for degradation [27]. It was reported that gene encoding ClpC protein was also vital in bile and acid tolerance [28].

Gene coding for protein DltB which modulates the immune response through D-alanylation of lipoteichoic acid was discerned in the *L. plantarum* PA21 genome. The lipoteichoic acid is a component of the bacterial cell wall that has been associated with pro-inflammatory interaction through Toll-like receptor 2 (TLR2) [29]. Therefore, the *dltB* gene is an important entity in the immunomodulatory effects in *L. plantarum* PA21. In addition, the *L. plantarum* PA21 probiotic characteristic was strengthened with the presence of adhesion genes coding for Msa, LamA and SrtA proteins. The adhesive property in probiotic strains is of paramount importance, by virtue of its ability to mediate adhesion to cells and prevent adhesion and invasion of pathogens [30]. Overall, the probiotic-associated genes in *L. plantarum* PA21 were mostly pleiotropic genes in which one gene could have multiple functions and tolerance against different stressors and may not just be restricted to a single function. Furthermore, these genes are likely to have an indirect impact on the postbiotic metabolite production by affecting its overall growth and metabolism.

To substantiate the results from genome analysis, the antimicrobial potential of CFS was tested on MRSA and *K. pneumoniae* clinical isolates. To achieve a state of parity, chloramphenicol, a broad-spectrum antibiotic, was used as the positive control. The results clearly indicated that CFS of *L. plantarum* PA21 was more selective against Gram-positive bacteria than Gram-negative bacteria. This was also evident in several other studies where the CFS from *Lactiplantibacillus* sp. showed greater inhibition against Gram-positive bacteria such as MRSA than Gram-negative bacteria such as *K. pneumoniae, Pseudomonas aeruginosa* and *Escherichia coli* [31, 32]. One possible justification to account for the occurrence of such narrow inhibition spectrum by *L. plantarum* PA21 might be attributable to the differences in the MDR isolates’ resistance profiles and/or the composition of its CFS. Such a narrow spectrum might also be linked to the predicted bacteriocins in the *L. plantarum* PA21 genome as they are generally more potent against Gram-positive pathogens than Gram-negative pathogens [33]. This stems from the relevance of a thick peptidoglycan layer in Gram-positive bacteria that is usually porous in contrast to the outer lipid membrane-protected peptidoglycan layer in Gram-negative bacteria that serves as a formidable barrier. Bacteriocins therefore easily surmount this circumstance by disrupting the cell membrane and forming pores in Gram-positive bacteria [34].

Following that, the effects of heat, pH, and enzymes on bacteriocin activity of *L. plantarum* PA21 was analysed against MRSA 10. The antimicrobial activity of *L. plantarum* PA21 CFS was relatively unaffected across a wide range of temperature. Interestingly, when CFS was treated with proteolytic enzymes, the antimicrobial activity was completely lost, indicating the presence of proteinaceous substances in the CFS. In contrast, no antimicrobial activity by *L. plantarum* PA21 CFS was observed from pH 5 onwards. Thus, it is speculated that the acidity of CFS besides bacteriocin is partly responsible for the inhibitory effect as antimicrobial activity drastically declined in parallel to the increase in pH. These results corroborated with previous findings on *L. plantarum* NTU102 CFS that similarly exhibited a narrow pH range (pH 1 to pH 4) with unrecorded activity at neutral and alkaline pH while antimicrobial activity when treated with proteolytic enzymes such as pepsin, proteinase K and trypsin, significantly reduced [35]. Similarly, when Qian et al. (2020) [36] neutralized the CFS of *L. plantarum* strains, a significant reduction in antimicrobial activity was observed against the tested pathogens. Nevertheless, the primary antimicrobial effect exerted by the *L. plantarum* PA21 CFS on the clinical isolates may likely be due to the production of a range of antimicrobials such as organic acids which reduces the overall pH of the environment in tandem with other bioactive compounds such as bacteriocins. Therefore, all findings support that *L. plantarum* PA21 postbiotic metabolites could contribute to the antimicrobial potential against MDR pathogens and are worth bioprospecting.

## Conclusions

An insight to the genome of *L. plantarum* PA21 revealed an assortment of bioactive compounds and probiotic features that could contribute to the antimicrobial properties against MDR pathogens. Whilst this study did not confirm the exact postbiotic metabolites involved, it did partially substantiate the presence of what would have been an array of metabolites that contributed to the antimicrobial activity of *K. pneumoniae* and MRSA clinical isolates. Ultimately, the insights gained on *L. plantarum* PA21 gene signatures and its regulatory mechanisms may be of assistance as potential antimicrobial agents in overcoming the AMR tide. Further research is necessary to fully characterise and elucidate the mechanisms underlying its various bioactivities against MDR pathogens and assess the safety and efficacy of *L. plantarum* PA21 in vitro for application as a postbiotic both as a standalone therapy or in combination with other treatments.

## Materials and methods

### Strains and growth conditions

The *L. plantarum* PA21 strain previously isolated from a tropical plant *Pandanus amaryllifolius* [37] has been deposited in the Microbial Culture Collection Unit (UNiCC) UPM (accession no: UPMC267) and was used as the test strain for postbiotic metabolite production while the MDR strains of *K. pneumoniae* and MRSA provided by Hospital Pengajar Universiti Putra Malaysia (HPUPM) were used as the indicator strains in this study. *L. plantarum* PA21 were grown in de Man, Rogosa and Sharpe (MRS) broth (Merck, Germany) at 37 °C for 24 h aerobically. The *K. pneumoniae* and MRSA strains were grown overnight in Luria Bertani (LB) broth and Brain Heart Infusion (BHI) broth, respectively at 37 °C for 24 h.

### Genomic DNA isolation and whole genome sequencing (WGS)

*L. plantarum* PA21 was cultivated overnight in MRS broth at 37 °C and overnight culture was centrifuged the next day at 12,000 rpm for five minutes to obtain bacterial cells. The genomic DNA of *L. plantarum* PA21 was isolated using the PrimeWay Genomic DNA extraction kit (1st BASE, Malaysia). DNA quality was checked using a spectrophotometer (260/230 > 1.8 and 260/280 > 2.0; Implen, Germany) and 1% agarose electrophoresis gel. The whole genome sequencing of *L. plantarum* PA21 was carried out using the MinION MK1C (Oxford Nanopore Technologies, UK) with a R9.4.1 flow cell at Nanyang Technological University, Singapore. Prior to sequencing, the DNA was barcoded along with other microbial DNA through the PCR barcoding process using Rapid PCR Barcoding Kit’s protocol (SQK-RPB004). Base calling was done using guppy v3.2.2 (https://timkahlke.github.io/LongRead_tutorials/BS_G.html*)* to produce fastq files using the base calling model of dna_r9.4.1_450bps_hac.cfg.

### Genome assembly and annotation

Demultiplexed fastq files were assembled using Flye (https://github.com/fenderglass/Flye*)*, a long-read assembler to produce contigs. Further correction of the draft sequences from Flye was done using Medaka (https://github.com/nanoporetech/medaka*).* The genome was annotated in Pathosystems Resource Integration Center (PATRIC) [38].

### Phylogenomic analysis and average nucleotide identity (ANI)

Phylogenomic analysis was done through GToTree pipeline [39]. In brief, representative genomes from the genus of *Lactiplantibacillus* and several complete genomes of *L. plantarum* were selected and downloaded using GToTree by listing the Refseq’s accession ID. The genome of *L. plantarum* PA21 and the outgroup, *Bacillus cereus* (accession: GCF_021655335.1) were included in the analysis by using the locally available fasta files. The ORF of these genomes were predicted using Prodigal v2.6.3 [40] and HMMsearch v3.3.2 [41] was used to search for an in-house set of HMM of Firmicutes. The outputs were then trimmed using trimAl v1.4.rev15 [42] and concatenated before parsing to Iqtree2 together with the partition file (http://www.iqtree.org) using ultrafast bootstrap approximation for phylogenetic estimation of maximum likelihood tree [43]. Taxonkit [44] was used to provide genus and species name of the input genome based on RefSeq accession ID. Tree was visualised using Interactive Tree of Life (itol) web server (https://itol.embl.de/*)* with branch length ignored to further visualise the clade separation.

Similar sets of genomes were subjected to ANI analysis using fastANI [45]. The heatmap and clustering were done using ANIclustermap (https://github.com/moshi4/ANIclustermap*).* Protologger version 1.0 was used mainly to infer environmental distribution utilizing the built-in Integrated Microbial NGS Platform (IMNGS) database of 1000 amplicon sequencing data [46, 47]. The output was parsed and visualized using matplotlib.

### Pangenome analysis

Pangenome analysis was done using the program Anvi’o version 8 [48]. In brief, the genomes from Clade 1 were used as the input genomes. The fasta files were first formatted using anvi-reformat-fasta program before being converted into individual contigs database using anvi-gen-contigs-database. The contigs database were annotated with functional annotation from Ncbi-cogs, Pfam, Kofam and CAZyme using anvi-run-ncbi-cogs, anvi-run-pfams, anvi-run-kegg-kofams and anvi-run-cazymes, respectively. The individual contigs database were then combined into a genome storage and were used to create a pangenome using anvi-gen-genome-storage and anvi-pan-genome. The pangenome was analyzed using anvi-display-pan and was summarized using anvi-summarize.

### Genome mining

The secondary metabolite prediction tools, BAGEL 4 [49] and antiSMASH [50] were mined to determine putative bacteriocin clusters and genes related to biosynthesis of antimicrobial proteins. The linear genome of *L. plantarum* PA21 mapped to putative bacteriocin clusters was constructed using ProKsee [51]. All tools were used at default settings. The image of gene operon was generated using DNA Features Viewer (https://github.com/Edinburgh-Genome-Foundry/DnaFeaturesViewer) based on BAGEL4 output.

### Determination of probiotic and virulence genes

The annotated genome was screened for similar probiotic genes through the NCBI database. The selection of probiotic related genes was based on a previous report that defined the genetic determinants involved in probiotic *Lactobacillus* sp [30]. BLASTP was used for the identification of genes with similar amino acid sequences using a default configuration. Then, the annotated genes of *L. plantarum* PA21 were analysed through the virulence factors database (VFDB) [52] using Abricate (https://github.com/tseemann/abricate*).*

### Antimicrobial activity

The antimicrobial activity by *L. plantarum* PA21 CFS was evaluated on clinical isolates through the agar well diffusion method described by Tagg and Mcgiven [53]. The overnight *L. plantarum* PA21 broth culture was centrifuged (4000 rpm, 10 min, 4 °C) to obtain cell-free supernatant (CFS). Briefly, 100 uL of CFS was pipetted into the wells (8–9 mm) of Mueller-Hinton (MH) agar seeded with indicator strains adjusted to an optical density (OD) of 0.08–0.1 at 595 nm. MRS broth only served as the negative control while antibiotic disk; chloramphenicol (30 ug) served as the positive control. The inhibition zones were measured after incubation at 37 °C for 24 h and the antimicrobial activity (AU) were reported as a unit area of the inhibition zone per unit volume sample loaded into well (mm2/mL) [54].

### Effect of pH, enzyme and temperature on *L. plantarum* PA21 CFS

To determine the effect of pH, the CFS of *L. plantarum* PA21 was adjusted to pH values ranging from 2.0 to 10.0 using 1 M HCL and 1 M NaOH. The proteinaceous nature of antimicrobial substances in CFS was determined by incubating the CFS with 1 mg/mL proteinase K and 1 mg/mL trypsin (Sigma-Aldrich, USA) at 37 °C for two hours. The effect of temperature was investigated by incubating the CFS at 30, 50, 70, 100 and 121 °C for 15 min. Antimicrobial activity was then determined through the agar well diffusion method. Untreated CFS was used as a control.

### Data analysis

The results in this study were presented as means ± standard deviation (*n* = 3). All statistical analysis was performed using GraphPad Prism 9 version 9.0 for Windows.

### Electronic supplementary material

Below is the link to the electronic supplementary material.

Supplementary Material 1

## Data Availability

The *L. plantarum* PA21 whole genome sequence reported in this study has been deposited at NCBI under Bioproject with the accession number PRJNA987396.

## Declarations

### Permission

We hereby confirm that the necessary permission to utilize *L. plantarum* PA21 strain isolated from *Pandanus amaryllifolius* in this study from Prof Raha Abdul Rahim has been obtained.

## Abbreviations

CFS: Cell-free supernatant
MDR: Multi-drug resistant bacteria
MRSA: Methicillin-resistant *Staphylococcus aureus*
ANI: Average nucleotide identity
BGC: Biosynthetic gene cluster
