# Supplementary material for: Genome mining of Lactiplantibacillus plantarum PA21: insights into its antimicrobial potential
Source: BMC Genomics. 2024 Jun 6;25:571. doi: 10.1186/s12864-024-10451-7 (PMC11157852; doi:10.1186/s12864-024-10451-7)
Supplement: Supplementary file 1 — Supplementary Material 1 [file 12864_2024_10451_MOESM1_ESM.docx]

**Genome Mining of *Lactiplantibacillus plantarum* PA21: Insights into Its Antimicrobial and Antibiofilm Potential**

Sharleen Livina Isaac ^1^, Ahmad Zuhairi Abdul Malek ^2^, Nurul Syafika Hazif ^1^, Farah Syahrain Roslan ^1^, Amalia Mohd Hashim ^1, 2^, Adelene Ai-Lian Song ^1^, Raha Abdul Rahim ^3,4^ & *Wan Nur Ismah Wan Ahmad Kamil^1^

^1^ *Department of Microbiology, Faculty of Biotechnology and Biomolecular Sciences, Universiti Putra Malaysia (UPM), 43400 Serdang, Selangor, Malaysia.*

^2^ *Halal Products Research Institute, Universiti Putra Malaysia (UPM), 43400 Serdang, Selangor, Malaysia.*

^3^ *Department of Cell and Molecular Biology, Faculty of Biotechnology and Biomolecular Sciences, Universiti Putra Malaysia (UPM) 43400 Serdang, Selangor, Malaysia.*

^4^ *National Institutes of Biotechnology Malaysia (NIBM), 43400 Serdang Selangor, Malaysia.*

**Corresponding author:* [wn_ismah@upm.edu.my](mailto:wn_ismah@upm.edu.my)

**Table S1.** The strain distribution of *Lactiplantibacillus* *plantarum*.

| **Accession ID** | **Strain** | **Isolation Source** | **Genome size (Mb)** |
| --- | --- | --- | --- |
| GCF_011304595.2 | LS7 | Human faeces | 3.4 |
| GCF_000466905.3 | 2025 | Milk | 3.5 |
| GCF_016775685.1 | S58 | Chinese pickle | 3.3 |
| GCF_016812075.1 | CXG9 | Stinky xiancaigeng | 3.4 |
| GCF_016598735.1 | PC518 | - | 3.5 |
| GCF_016838645.1 | KM2 | Ripening beef | 3.4 |
| GCF_016894405.1 | Lp900 | Ogi (red sorghum) | 3.4 |
| GCF_017068235.1 | GR0128 | Fu-Tsai | 3.3 |
| GCF_017301935.1 | 12 | *Scophthalmus maximus* | 3.3 |
| GCF_017351995.1 | AR195 | Rice wine rice syrup | 3.4 |
| GCF_017576965.1 | KLDS1.0386 | Fermented Mongolian dairy | 3.3 |
| GCF_017742875.1 | LRCC5314 | Kimchi | 3.2 |
| GCF_017798305.1 | NCIMB8826 | - | 3.3 |
| GCF_018351295.1 | ATCC 202195 | Infant faeces | 3.4 |
| GCF_018588615.2 | subsp *plantarum* (M17) | Motal cheese | 3.3 |
| GCF_018588605.2 | subsp *plantarum* (M19) | Motal cheese | 3.5 |
| GCF_018588665.2 | subsp *plantarum* (M8) | Motal cheese | 3.3 |
| GCF_019076805.1 | XJ25 | Wine | 3.2 |
| GCF_019211765.1 | subsp *plantarum* (GR1184) | Food | 3.3 |
| GCF_019211785.1 | subsp *plantarum* (GR1187) | Food | 3.3 |
| GCF_019399915.1 | 41P | Meat | 3.3 |
| GCF_019321805.1 | L75a | *Scylla seratta* | 3.4 |
| GCF_019469465.1 | MSD1 | Curd | 3.1 |
| GCF_019425695.1 | DW12 | Fermented food | 3.2 |
| GCF_021279005.2 | 022AE | Fermented dairy | 3.2 |
| GCF_020881935.1 | ZW5 | Water | 3.4 |
| GCF_021559915.1 | A8 | Animal dropping | 3.2 |
| GCF_021559675.1 | P9 | Plant | 3.4 |
| GCF_021560135.1 | W2 | *Penaeus vannamei* | 3.3 |
| GCF_021650875.1 | subsp *plantarum* (GR1186) | Food | 3.3 |
| GCF_022810685.1 | SCB0151 | Kefir grain | 3.2 |
| GCF_022558425.1 | ST | De’ang pickled tea | 3.3 |
| GCF_023278325.1 | MNCW 1 | Fermented food | 3.3 |
| GCF_023207995.1 | LP-F1 | Fermented milk | 3.3 |
| GCF_023347215.1 | VHProbi V38 | Kimchi soup | 3.2 |
| GCF_023348385.1 | LPC904 | Human gut | 3.3 |
| GCF_023348465.1 | LPIMC513 | Human faeces | 3.2 |
| GCF_023348525.1 | LPT52 | Raw milk cheese | 3.3 |
| GCF_023370155.1 | JB-1 | Fermented sausage | 3.3 |
| GCF_023973045.1 | Z.6-1 | Dairy starter culture | 3.3 |
| GCF_024137845.1 | DM083 | Human tongue coating | 3.2 |
| GCF_024137985.1 | 44929 | Pickle | 3.5 |
| GCF_024181685.1 | P9 | Sour porridge | 3.3 |
| GCF_024396815.1 | HOM3204 | Pickled cabbage | 3.3 |
| GCF_024181705.1 | Lp-6 | Kefir | 3.5 |
| GCF_024442115.1 | LpYC41 | Pickle | 3.3 |
| GCF_024732385.1 | Q180 | Human faeces | 3.2 |
| GCF_024758665.1 | VHProbi O04 | - | 3.3 |
| GCF_024758745.1 | VHProbi O10 | - | 3.3 |
| GCF_024800605.1 | SRCM210459 | Pig faeces | 3.3 |
| GCF_024969905.1 | SRCM210465 | Pig faeces | 3.3 |
| GCF_024969715.1 | SRCM210576 | Cattle faeces | 3.3 |
| GCF_024970125.1 | SRCM210579 | Cattle faeces | 3.3 |
| GCF_024970145.1 | SRCM210580 | Cattle faeces | 3.3 |
| GCF_024970165.1 | SRCM210797 | Cattle faeces | 3.2 |
| GCF_025144505.1 | HMX2 | Northeast sauerkraut | 3.3 |
| GCF_025402835.1 | MGEL20154 | Kimchi | 3.2 |
| GCF_025723165.1 | BF 15 | Breast-fed infant faeces | 3.3 |
| GCF_026013765.1 | E2 | *Larimichthys crocea* | 3.5 |
| GCF_026016545.1 | VHProbi P06 | Kimchi | 3.4 |
| GCF_026183415.1 | BRD L15 | Dog stool | 3.2 |
| GCF_026153115.1 | L55 | Kimchi | 3.4 |
| GCF_027474465.1 | LL441 | Traditional cheese | 3.2 |
| GCF_026240755.1 | Y42 | Fish | 3.3 |
| GCF_026689375.1 | BC015 | Chinese sauerkraut | 3.4 |
| GCF_027557615.1 | FLPL05 | Human faeces | 3.3 |
| GCF_027920405.1 | ZW59 | Gut | 3.2 |
| GCF_027558615.1 | VHProbi E15 | Human | 3.3 |
| GCF_028463965.1 | MWLp-12 | Human | 3.3 |
| GCF_028201575.1 | P6 | Breast milk | 3.3 |
| GCF_028768485.1 | SMB758 | Kimchi | 3.2 |
| GCF_028869445.1 | VHProbi V22 | Human | 3.4 |
| GCF_029543005.1 | KF511 | Kimchi | 3.3 |
| GCF_029542245.1 | EN6 | Phyllosphere endophyte | 3.3 |
| GCF_029590535.1 | ZFM518 | Infant faeces | 3.3 |


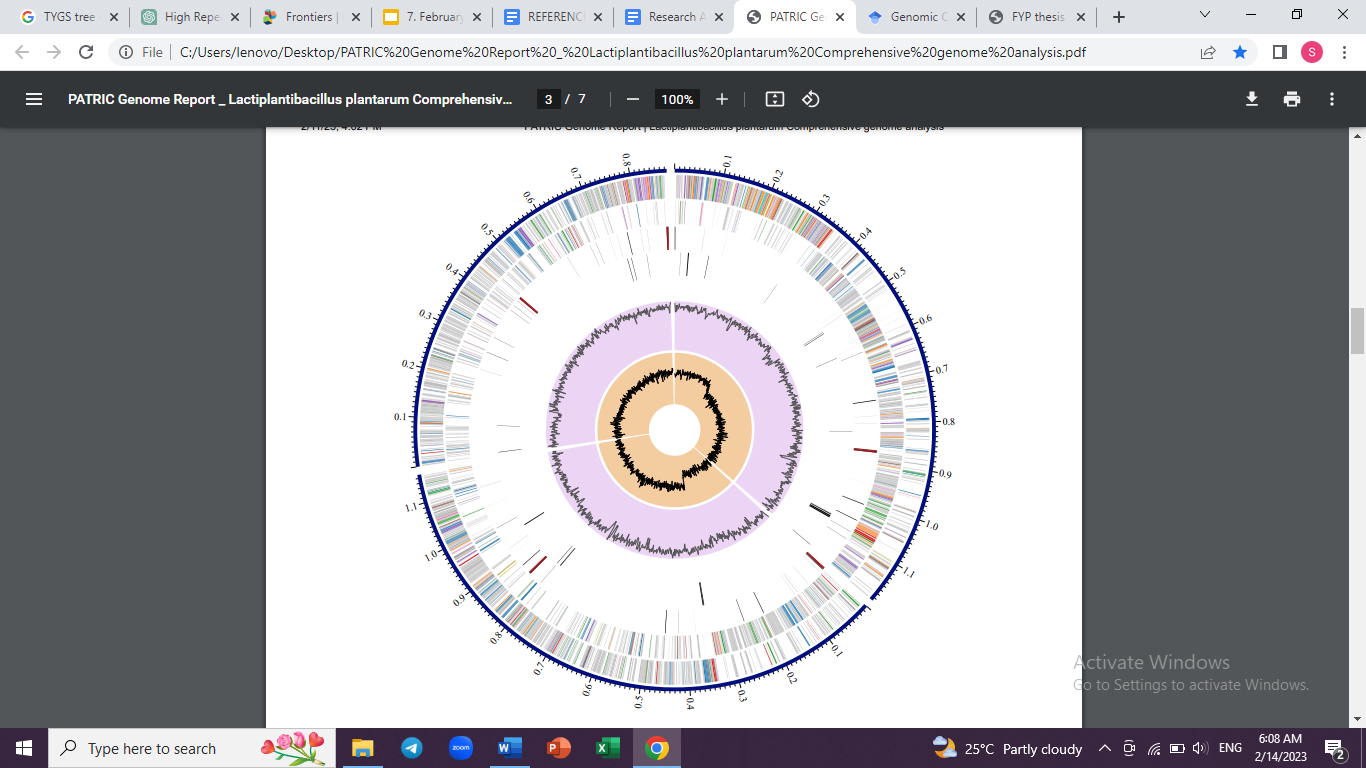


**Fig.S1 The genome sequence of *L. plantarum* PA21 annotated through the PATRIC database.** Circular genome characteristics of *L. plantarum* PA21. From outer to inner rings, the genomic features show the contigs, CDS on the forward strand, CDS on the reverse strand, RNA genes, CDS with homology to know virulence factors, GC content and GC skew. The colours of the CDS on the forward and reverse strand indicate the subsystems that each gene belongs to.

**Table.S2** The prevalence and mean relative abundance of L. plantarum across different environments.

| **Metagenome** | **Prevalamce** | **Mean relative abundance** | **Standard deviation** |
| --- | --- | --- | --- |
| Marine metagenome | 0.20% | 0.00% | 0.00%. |
| Activated sludge metagenome | 6.40% | 0.01% | 0.01%. |
| Human skin metagenome | 5.10% | 0.05% | 0.14%. |
| Chicken gut metagenome | 1.40% | 0.03% | 0.06%. |
| Freshwater metagenome | 2.00% | 0.04% | 0.12%. |
| Wastewater metagenome | 15.90% | 0.09% | 0.63%. |
| Human vaginal metagenome | 2.80% | 0.98% | 1.76%. |
| Plant metagenome | 2.50% | 1.09% | 4.89%. |
| Mouse gut metagenome | 2.00% | 1.22% | 2.14%. |
| Bovine gut metagenome | 1.10% | 0.00% | 0.00%. |
| Human gut metagenome | 2.90% | 0.30% | 0.84%. |
| Soil metagenome | 0.60% | 0.02% | 0.02%. |
| Pig gut metagenome | 9.60% | 0.72% | 3.09%. |
| Insect gut metagenome | 4.00% | 1.75% | 3.05%. |
| Human lung metagenome | 1.10% | 0.01% | 0.00%. |
| Human oral metagenome | 2.10% | 0.05% | 0.08%. |
| Coral metagenome | 0.50% | 0.09% | 0.13%. |
| Marine sediment metagenome | 0.40% | 0.03% | 0.04%. |
| Rhizosphere metagenome | 0.40% | 0.00% | 0.00%. |

**Table S3.** The singletons gene cluster of *L. plantarum* PA21.

| gene_cluster_id | COG20_CATEGORY | Pfam_ACC | Pfam | COG20_FUNCTION | CAZyme |  |  |  |
| --- | --- | --- | --- | --- | --- | --- | --- | --- |
| GC_00004419 |  | PF00144.28 | Beta-lactamase |  |  |  |  |  |
| GC_00004401 | Replication, recombination and repair | PF00270.33 | DEAD/DEAH box helicase | Superfamily II DNA helicase RecQ (RecQ) (PDB:1OYW) |  |  |  |  |
| GC_00004096 | Carbohydrate transport and metabolism | PF00535.30 | Glycosyl transferase family 2 | Glycosyltransferase, GT2 family (WcaE) (PDB:2Z86) | GT2_Glycos_transf_2.hmm |  |  |  |
| GC_00004330 | Cell wall/membrane/envelope biogenesis | PF00535.30 | Glycosyl transferase family 2 | Glycosyltransferase involved in cell wall bisynthesis (WcaA) (PDB:5MLZ) | GT2_Glycos_transf_2.hmm |  |  |  |
| GC_00004350 |  | PF01381.26 | Helix-turn-helix |  |  |  |  |  |
| GC_00003865 | Defense mechanisms | PF01420.23 | Type I restriction modification DNA specificity domain | Restriction endonuclease S subunit (HsdS) (PDB:1YDX) |  |  |  |  |
| GC_00004249 |  | PF01726.20 | LexA DNA binding domain |  |  |  |  |  |
| GC_00003936 |  | PF02556.18 | Preprotein translocase subunit SecB |  |  |  |  |  |
| GC_00004164 | Replication, recombination and repair | PF03796.19 | DnaB-like helicase C terminal domain | Replicative DNA helicase (DnaB) (PDB:1B79) |  |  |  |  |
| GC_00004537 | Defense mechanisms | PF03819.21 | MazG nucleotide pyrophosphohydrolase domain | NTP pyrophosphatase, house-cleaning of non-canonical NTPs (MazG) (PDB:1VMG) |  |  |  |  |
| GC_00004293 |  | PF04404.16 | ERF superfamily |  |  |  |  |  |
| GC_00003806 |  | PF06030.16 | Bacterial protein of unknown function (DUF916) |  |  |  |  |  |
| GC_00004477 |  | PF06356.15 | Protein of unknown function (DUF1064) |  |  |  |  |  |
| GC_00004469 |  | PF10651.13 | BppU N-terminal domain |  |  |  |  |  |
| GC_00004528 |  | PF11023.12 | Zinc-ribbon containing domain |  |  |  |  |  |
| GC_00004127 | Cell wall/membrane/envelope biogenesis | PF11380.12 | Stealth protein CR2, conserved region 2 | Glycosyltransferase involved in cell wall bisynthesis (RfaB) (PDB:2IV7) |  |  |  |  |
| GC_00003892 |  | PF13384.10 | Homeodomain-like domain |  |  |  |  |  |
| GC_00004453 |  | PF13612.10 | Transposase DDE domain |  |  |  |  |  |
| GC_00004302 |  | PF13730.10 | Helix-turn-helix domain |  |  |  |  |  |
| GC_00004531 |  | PF14897.10 | EpsG family |  |  |  |  |  |
| GC_00004228 | Transcription | PF17726.5 | Dam-replacing HTH domain | DNA-directed RNA polymerase, sigma subunit (sigma70/sigma32) (RpoD) (PDB:1SIG) |  |  |  |  |
| GC_00003917 |  | PF19791.3 | Family of unknown function (DUF6275) |  |  |  |  |  |
| GC_00003816 | Defense mechanisms | PF20473.2 | MmeI, DNA-methyltransferase domain | Type II restriction/modification system, endonuclease and methylase domains (YeeA) (PDB:5HR4) |  |  |  |  |
| GC_00003953 | Cell cycle control, cell division, chromosome partitioning |  |  | Fe-S cluster carrier ATPase, Mrp/ApbC/NBP35 family (Mrp)  (PDB:2PH1) (PUBMED:18616280;19114487;31709520) |  |  |  |  |
| GC_00003828 |  |  |  |  |  |  |  |  |
| GC_00003885 |  |  |  |  |  |  |  |  |
| GC_00004002 |  |  |  |  |  |  |  |  |
| GC_00004031 |  |  |  |  |  |  |  |  |
| GC_00004045 |  |  |  |  |  |  |  |  |
| GC_00004107 |  |  |  |  |  |  |  |  |
| GC_00004122 |  |  |  |  |  |  |  |  |
| GC_00004163 |  |  |  |  |  |  |  |  |
| GC_00004188 |  |  |  |  |  |  |  |  |
| GC_00004210 |  |  |  |  |  |  |  |  |
| GC_00004218 |  |  |  |  |  |  |  |  |
| GC_00004251 |  |  |  |  |  |  |  |  |
| GC_00004269 |  |  |  |  |  |  |  |  |
| GC_00004338 |  |  |  |  |  |  |  |  |
| GC_00004342 |  |  |  |  |  |  |  |  |
| GC_00004349 |  |  |  |  |  |  |  |  |
| GC_00004417 |  |  |  |  |  | |  |  |
| GC_00004512 |  |  |  |  |  | |  |  |
| GC_00004533 |  |  |  |  | | | | GH43_18.hmm |
| GC_00004557 |  |  |  |  | | | |  |

**Table S4.** Enriched COG20 functions between cluster A and cluster B

| COG20_FUNCTION | enrichment_score | unadjusted_p_value | adjusted_q_value | associated_groups | accession | gene_clusters_ids | p_cluster_A | p_cluster_B |
| --- | --- | --- | --- | --- | --- | --- | --- | --- |
| 5-methylthioribulose/5-deoxyribulose/Fuculose 1-phosphate aldolase (methionine salvage, sugar degradation) (AraD) (PDB:1FUA) (PUBMED:31950558) | 16.00037433 | 6.33E-05 | 0.022499 | cluster_B | COG0235 | GC_00002807, GC_00002978, GC_00004421 | 0 | 1 |
| Superfamily I DNA and/or RNA helicase (DNA2) (PDB:2GJK) | 16.00037433 | 6.33E-05 | 0.022499 | cluster_B | COG1112 | GC_00002751 | 0 | 1 |
| ADP-ribosylglycohydrolase (DraG) (PDB:1T5J) | 12.44444444 | 4.19E-04 | 0.022499 | cluster_A | COG1397 | GC_00002824 | 1 | 0.1111 |
| Molybdopterin biosynthesis enzyme MoaB/MogA (MoaB) (PDB:1DI6) (PUBMED:32239579) | 12.44444444 | 4.19E-04 | 0.022499 | cluster_B | COG0521 | GC_00002827 | 0 | 8.89E-01 |
| GTP 3',8-cyclase (molybdenum cofactor biosynthesis protein MoaA) (MoaA) (PDB:1TV7) | 12.44444444 | 4.19E-04 | 0.022499 | cluster_B | COG2896 | GC_00002818 | 0 | 0.8889 |
| ABC-type polysaccharide transport system, permease component (LplB) (PDB:4TQU) | 12.44444444 | 4.19E-04 | 0.022499 | cluster_B | COG4209 | GC_00002843 | 0 | 0.8889 |
| Meiotically up-regulated gene 157 (Mug157) protein (function unknown) (PDB:2NVP) | 12.44444444 | 4.19E-04 | 0.022499 | cluster_B | COG3538 | GC_00002859 | 0 | 0.8889 |
| Cupin domain protein related to quercetin dioxygenase (QdoI) (PDB:2H0V)!!!Two-component response regulator, YesN/AraC family, consists of REC and AraC-type DNA-binding domains (YesN) | 12.44444444 | 4.19E-04 | 0.022499 | cluster_B | COG1917!!!COG4753 | GC_00002852 | 0 | 0.8889 |
| Molybdopterin synthase catalytic subunit MoaE (MoaE) (PDB:1FM0) (PUBMED:32239579)!!!Molybdopterin synthase sulfur carrier subunit MoaD (MoaD) (PDB:1FM0) (PUBMED:32239579) | 12.44444444 | 4.19E-04 | 0.022499 | cluster_B | COG0314!!!COG1977 | GC_00002793 | 0 | 0.8889 |
| PAS domain (PAS) (PDB:2MWG)!!!Signal transduction histidine kinase ComP (ComP) (PDB:4GT8) | 12.44444444 | 4.19E-04 | 0.022499 | cluster_B | COG2202!!!COG4585 | GC_00002864 | 0 | 0.8889 |
| Molybdopterin synthase catalytic subunit MoaE (MoaE) (PDB:1FM0) (PUBMED:32239579) | 12.44444444 | 4.19E-04 | 0.022499 | cluster_B | COG0314 | GC_00002789 | 0 | 0.8889 |
| N-acetyl-beta-hexosaminidase (Chb) (PDB:3RPM) (PUBMED:18063573) | 12.44444444 | 4.19E-04 | 0.022499 | cluster_B | COG3525 | GC_00002829 | 0 | 0.8889 |
| Nitrate reductase alpha subunit (NarG) (PDB:1Q16) | 12.44444444 | 4.19E-04 | 0.022499 | cluster_B | COG5013 | GC_00002835 | 0.00E+00 | 0.8889 |
| Nitrate reductase beta subunit (NarY) (PDB:1Y4Z) | 12.44444444 | 4.19E-04 | 0.022499 | cluster_B | COG1140 | GC_00002797 | 0.00E+00 | 0.8889 |
| Preprotein translocase subunit SecB (SecB) (PDB:1OZB) | 12.44444444 | 4.19E-04 | 0.022499 | cluster_B | COG1952 | GC_00002853 | 0.00E+00 | 0.8889 |
| Uncharacterized membrane protein YesL (YesL) | 12.44444444 | 4.19E-04 | 0.022499 | cluster_B | COG5578 | GC_00002795 | 0 | 0.8889 |
| Molybdopterin-guanine dinucleotide biosynthesis protein (MobB) (PDB:1NP6) (PUBMED:32239579) | 12.44444444 | 4.19E-04 | 0.022499 | cluster_B | COG1763 | GC_00002841 | 0 | 8.89E-01 |
| Molybdopterin-guanine dinucleotide biosynthesis protein A (MobA) (PDB:1E5K) (PUBMED:32239579) | 12.44444444 | 4.19E-04 | 0.022499 | cluster_B | COG0746 | GC_00002825 | 0 | 0.8889 |
| Signal transduction protein containing GAF and PtsI domains (PtsP) | 12.44444444 | 4.19E-04 | 0.022499 | cluster_B | COG3605 | GC_00002846 | 0 | 0.8889 |
| L-rhamnose isomerase (RhaA) (PDB:1D8W) | 12.44444444 | 4.19E-04 | 0.022499 | cluster_B | COG4806 | GC_00002794 | 0 | 0.8889 |
| Two-component response regulator, YesN/AraC family, consists of REC and AraC-type DNA-binding domains (YesN) | 12.44444444 | 4.19E-04 | 0.022499 | cluster_B | COG4753 | GC_00002805 | 0 | 0.8889 |
| Molybdenum cofactor biosynthesis enzyme MoaC (MoaC) (PDB:1EKR) (PUBMED:32239579) | 12.44444444 | 4.19E-04 | 0.022499 | cluster_B | COG0315 | GC_00002845 | 0 | 0.8889 |
| Nitrate reductase gamma subunit (NarI) (PDB:1Y5L) | 12.44444444 | 4.19E-04 | 0.022499 | cluster_B | COG2181 | GC_00002840 | 0.00E+00 | 0.8889 |
| L-rhamnose mutarotase (RhaM) (PDB:1X8D) | 12.44444444 | 4.19E-04 | 0.022499 | cluster_B | COG3254 | GC_00002809 | 0 | 0.8889 |
| Molybdopterin Mo-transferase (molybdopterin biosynthesis) (MoeA) (PDB:2NQU) (PUBMED:18801996;32239579) | 12.44444444 | 4.19E-04 | 0.022499 | cluster_B | COG0303 | GC_00002822 | 0 | 8.89E-01 |
| Nitrate reductase assembly protein NarJ, required for insertion of molybdenum cofactor (NarJ) | 12.44444444 | 4.19E-04 | 0.022499 | cluster_B | COG2180 | GC_00002823 | 0 | 0.8889 |
| Putative alpha-1,2-mannosidase (PDB:2WVX) | 12.44444444 | 4.19E-04 | 0.022499 | cluster_B | COG3537 | GC_00002848 | 0 | 0.8889 |
| Protein involved in initiation of plasmid replication (PDB:2Z9O) | 12.34288814 | 4.43E-04 | 0.022908 | cluster_A | COG5527 | GC_00002652 | 0.8571 | 0 |

**Table S5.** Enriched KOfam between cluster A and cluster B.

| KOfam | enrichment_score | unadjusted_p_value | adjusted_q_value | associated_groups | accession | gene_clusters_ids | p_cluster_A | p_cluster_B |
| --- | --- | --- | --- | --- | --- | --- | --- | --- |
| GntR family transcriptional regulator, arabinose operon transcriptional repressor | 16.00037 | 6.33E-05 | 0.01779 | cluster_B | K02103 | GC_00002833, GC_00002834, GC_00002964 | 0 | 1 |
| HTH-type transcriptional regulator, pheromone-responsive regulator | 12.44444 | 4.19E-04 | 0.01779 | cluster_A | K20375 | GC_00002869 | 1 | 0.1111 |
| MFS transporter, NNP family, nitrate/nitrite transporter | 12.44444 | 4.19E-04 | 0.01779 | cluster_B | K02575 | GC_00002781 | 0 | 0.8889 |
| molybdopterin adenylyltransferase [EC:2.7.7.75] | 12.44444 | 4.19E-04 | 0.01779 | cluster_B | K03638 | GC_00002827 | 0 | 0.8889 |
| L-rhamnose mutarotase [EC:5.1.3.32] | 12.44444 | 4.19E-04 | 0.01779 | cluster_B | K03534 | GC_00002809 | 0 | 0.8889 |
| iron complex transport system substrate-binding protein | 12.44444 | 4.19E-04 | 0.01779 | cluster_B | K02016 | GC_00002837 | 0 | 0.8889 |
| nitrate reductase gamma subunit [EC:1.7.5.1 1.7.99.-] | 12.44444 | 4.19E-04 | 0.01779 | cluster_B | K00374 | GC_00002840 | 0 | 0.8889 |
| nitrate reductase gamma subunit [EC:1.7.5.1 1.7.99.-] | 12.44444 | 4.19E-04 | 0.01779 | cluster_B | K03071 | GC_00002853, GC_00003936 | 0 | 0.8889 |
| two-component system, NarL family, sensor histidine kinase NreB [EC:2.7.13.3] | 12.44444 | 4.19E-04 | 0.01779 | cluster_B | K07683 | GC_00002864 | 0 | 0.8889 |
| molybdopterin-guanine dinucleotide biosynthesis adapter protein | 12.44444 | 4.19E-04 | 0.01779 | cluster_B | K03753 | GC_00002841 | 0 | 0.8889 |
| L-rhamnose isomerase [EC:5.3.1.14] | 12.44444 | 4.19E-04 | 0.01779 | cluster_B | K01813 | GC_00002794 | 0 | 0.8889 |
| sulfur-carrier protein | 12.44444 | 4.19E-04 | 0.01779 | cluster_B | K03636 | GC_00002793 | 0 | 0.8889 |
| GTP 3',8-cyclase [EC:4.1.99.22] | 12.44444 | 4.19E-04 | 0.01779 | cluster_B | K03639 | GC_00002818 | 0 | 0.8889 |
| putative aldouronate transport system substrate-binding protein | 12.44444 | 4.19E-04 | 0.01779 | cluster_B | K17318 | GC_00002791 | 0 | 0.8889 |
| two-component system, NarL family, response regulator NreC | 12.44444 | 4.19E-04 | 0.01779 | cluster_B | K07696 | GC_00002788 | 0 | 0.8889 |
| molybdopterin synthase catalytic subunit [EC:2.8.1.12] | 12.44444 | 4.19E-04 | 0.01779 | cluster_B | K03635 | GC_00002789 | 0 | 0.8889 |
| two-component system, sensor histidine kinase YesM [EC:2.7.13.3] | 12.44444 | 4.19E-04 | 0.01779 | cluster_B | K07718 | GC_00002799 | 0 | 0.8889 |
| two-component system, response regulator YesN | 12.44444 | 4.19E-04 | 0.01779 | cluster_B | K07720 | GC_00002805 | 0 | 0.8889 |
| molybdopterin molybdotransferase [EC:2.10.1.1] | 12.44444 | 4.19E-04 | 0.01779 | cluster_B | K03750 | GC_00002822 | 0 | 0.8889 |
| putative aldouronate transport system permease protein | 12.44444 | 4.19E-04 | 0.01779 | cluster_B | K17320 | GC_00002811, GC_00002843 | 0 | 0.8889 |
| iron complex transport system permease protein | 12.44444 | 4.19E-04 | 0.01779 | cluster_B | K02015 | GC_00002831 | 0 | 0.8889 |
| hexosaminidase [EC:3.2.1.52] | 12.44444 | 4.19E-04 | 0.01779 | cluster_B | K12373 | GC_00002829 | 0 | 0.8889 |
| nitrate reductase / nitrite oxidoreductase, alpha subunit [EC:1.7.5.1 1.7.99.-] | 12.44444 | 4.19E-04 | 0.01779 | cluster_B | K00370 | GC_00002835 | 0 | 0.8889 |
| rhamnulokinase [EC:2.7.1.5] | 12.44444 | 4.19E-04 | 0.01779 | cluster_B | K00848 | GC_00002792 | 0 | 0.8889 |
| rhamnulose-1-phosphate aldolase [EC:4.1.2.19] | 12.44444 | 4.19E-04 | 0.01779 | cluster_B | K01629 | GC_00002807 | 0 | 0.8889 |
| nitrate reductase / nitrite oxidoreductase, beta subunit [EC:1.7.5.1 1.7.99.-] | 12.44444 | 4.19E-04 | 0.01779 | cluster_B | K00371 | GC_00002797 | 0 | 0.8889 |
| nitrogen regulatory protein A | 12.44444 | 4.19E-04 | 0.01779 | cluster_B | K10851 | GC_00002846 | 0 | 0.8889 |
| nitrate reductase molybdenum cofactor assembly chaperone NarJ/NarW | 12.44444 | 4.19E-04 | 0.01779 | cluster_B | K00373 | GC_00002823 | 0 | 0.8889 |
| cyclic pyranopterin monophosphate synthase [EC:4.6.1.17] | 12.44444 | 4.19E-04 | 0.01779 | cluster_B | K03637 | GC_00002845 | 0 | 0.8889 |
| molybdenum cofactor guanylyltransferase [EC:2.7.7.77] | 12.44444 | 4.19E-04 | 0.01779 | cluster_B | K03752 | GC_00002825 | 0 | 0.8889 |

**Table S6.** Enriched KEGG modules between cluster A and cluster B.

| KEGG_Module | enrichment_score | unadjusted_p_value | adjusted_q_value | associated_groups | accession | gene_clusters_ids | p_cluster_B | p_cluster_A |
| --- | --- | --- | --- | --- | --- | --- | --- | --- |
| Molybdenum cofactor biosynthesis, GTP => molybdenum cofactor | 12.44444 | 4.19E-04 | 0.012158 | cluster_B | M00880 | GC_00002789, GC_00002818, GC_00002822, GC_00002827, GC_00002845 | 0.8889 | 0 |
| Keratan sulfate degradation | 12.44444 | 4.19E-04 | 0.012158 | cluster_B | M00079 | GC_00002829 | 0.8889 | 0 |
| Nitrate assimilation | 12.44444 | 4.19E-04 | 0.012158 | cluster_B | M00615 | GC_00002781 | 0.8889 | 0 |
| Dissimilatory nitrate reduction, nitrate => ammonia!!!Denitrification, nitrate => nitrogen | 12.44444 | 4.19E-04 | 0.012158 | cluster_B | M00530!!!M00529 | GC_00002840 | 0.8889 | 0 |
| Dissimilatory nitrate reduction, nitrate => ammonia!!!Denitrification, nitrate => nitrogen!!!Complete nitrification, comammox, ammonia => nitrite => nitrate | 12.44444 | 4.19E-04 | 0.012158 | cluster_B | M00530!!!M00529!!!M00804 | GC_00002797, GC_00002835 | 0.8889 | 0 |
| Entner-Doudoroff pathway, glucose-6P => glyceraldehyde-3P + pyruvate!!!Semi-phosphorylative Entner-Doudoroff pathway, gluconate => glycerate-3P!!!D-Galacturonate degradation (bacteria), D-galacturonate => pyruvate + D-glyceraldehyde 3P!!!D-Glucuronate degradation, D-glucuronate => pyruvate + D-glyceraldehyde 3P | 9.679015 | 0.001864 | 0.045043 | cluster_B | M00008!!!M00308!!!M00631!!!M00061 | GC_00002916 | 0.7778 | 0 |
